# Perceptions of medical students and their facilitators on clinical communication skills teaching, learning, and assessment

**DOI:** 10.3389/fpubh.2023.1168332

**Published:** 2023-06-26

**Authors:** Sari Puspa Dewi, Amanda Wilson, Robbert Duvivier, Brian Kelly, Conor Gilligan

**Affiliations:** ^1^Department of Public Health, Faculty of Medicine, Universitas Padjadjaran, Bandung, Indonesia; ^2^School of Medicine and Public Health, The University of Newcastle, Callaghan, NSW, Australia; ^3^School of Nursing and Midwifery, The University of Technology Sydney, Sydney, NSW, Australia; ^4^Parnassia Psychiatric Institute, The Hague, Netherlands; ^5^Centre for Education Development and Research in Health Professions (CEDAR), Faculty of Medical Sciences, University Medical Centre Groningen, University of Groningen, Groningen, Netherlands

**Keywords:** clinical communication skills, learning environment, perception, medical student, facilitator

## Abstract

**Introduction:**

Despite various efforts to develop communication skills (CS) in the classroom, the transfer of these skills into clinical practice is not guaranteed. This study aimed to identify barriers and facilitators of transferring CS from the classroom to clinical environments.

**Methods:**

A qualitative study was conducted at one Australian medical school to explore the experiences and perceptions of facilitators and students in relation to teaching and learning clinical CS. Thematic analysis was used to analyze data.

**Results:**

Twelve facilitators and sixteen medical students participated in semi-structured interviews and focus-group discussions, respectively. Primary themes included the value of teaching and learning, alignment between approaches to teaching and actual clinical practices and students’ perceptions of practice, and challenges in different learning environments.

**Discussion:**

This study reinforces the value of teaching and learning CS by facilitators and students. Classroom learning provides students with a structure to use in communicating with real patients, which can be modified to suit various situations. Students have limited opportunities, however, to be observed and receive feedback on their real-patient encounters. Classroom session that discussed CS experiences during clinical rotation is recommended to strengthen learning both the content and process of CS as well as transitioning to the clinical environment.

## 1. Introduction

Effective communication can positively impact patient health outcomes. Studies report that depressive disorders (1), physiologic measurements such as blood pressure in hypertensive patients and blood sugar level in diabetic patients (2), as well as headache symptoms (3), are affected by effective verbal and nonverbal communication by doctors (4). Over recent decades, training in interpersonal communication has become increasingly embedded into medical education, with its inclusion as a primary competency in medical councils and program accreditation bodies internationally (5–8). As a result of the inclusion of communication in the World Federation of Medical Education (WFME) basic standards of medical education (9), along with individual country accreditation requirements, every medical school is required to incorporate communication skills teaching and learning as an integral part of the medical curriculum (8, 10).

Experts agree that communication skills can be effectively taught and learnt (11, 12). Clinical communication skills are a complex combination of clinical reasoning skills (the knowledge), process skills (how to deliver the knowledge) and perceptual skills (recognizing emotions and meanings in the communication) (13). These skills are interrelated, and it is recommended that they are taught and learned as an integrated process (14). Separating these skills in medical education has been found to be detrimental to the medical interviewing process (15).

Studies show that most efforts to teach communication skills to medical students have positive impacts on at least some communication skill outcomes, with training that incorporates feedback likely to have the most impact (16). The most common methods applied in teaching and learning communication skills for medical students are small group and experiential methods (17), often using role-play with simulated patients or peers in the classroom setting. In the clinical environment, communication skills are learnt through informal teachings, such as observing clinicians communicating with patients or students taking patient histories by themselves and reporting to the clinical facilitators (16).

In the clinical environment, students rely more heavily on indirect teaching through the observation of clinicians (17, 18). This places greater emphasis on the importance of role-models and the risks associated with the hidden curriculum. The hidden curriculum refers to the social norms and behaviors that exist but which are not usually explicitly acknowledged in learning environments (19). When the behaviors observed in clinical environments do not align with or even directly contradict the learning promoted in more structured teaching settings, students are receiving inconsistent messages. In addition, interactions with real patients are rarely observed or evaluated (20). In the context of learning about clinical communication skills, students observing different communication approaches modeled by clinical facilitators brings confusion on the importance of communication that is applied to patients (17, 18). The educational conditions in clinical environments are often considered inadequate for learning, particularly with regard to chaotic environments and lack of supervision due to clinical workload and time constraints of those teaching in clinical environments (18). Furthermore, students receive feedback that focuses on the clinical content rather than the process of communication, which can convey that communication skills are less necessary than medical knowledge (21, 22).

Students’ communication skills are commonly assessed using observation of students and patient interaction, with real patients in clinical practice or simulated patients in structured environments during Objective Structured Clinical Examination (OSCE) (21). In this type of assessment, students’ communication skills behaviors are observed against a standard checklist. Recent reviews show that OSCEs remain widely used and generally apply good assessment practices such as blueprinting to curricula and the use of valid and reliable instruments (23, 24). Therefore, this OSCE is suggested to be more suitable to evaluate a more junior level of training (21).

Whilst communication skills learning in medical students are well-addressed, there remains a challenge in the transfer of knowledge and skills from the classroom to clinical practice (25–29). The structured approaches to communication are often taught in the classroom setting, however, students report difficulties applying these structures and skills in clinical environments (29–33). The transition from primarily classroom-based learning in the early years of training to learning in clinical environments is associated with a range of changes due to different conditions; heavy workloads and long working hours, different teaching and assessment methods, the uncertainty of students’ roles, and adaptation to a more self-directed learning style (30, 34). These findings indicated that classroom skills, suggested structures and processes are not always applied in the clinical environment (18, 32, 35).

While the importance of clinical communication skills teaching and learning is well established, less is known about how, and to what extent, students, and facilitators, who are involved in teaching communication skills, address communication skills as part of medical training in clinical contexts. Understanding the value of these teaching and learning methods from the perspective of both those who learn the skills and facilitate the learning is important to strengthen learning these skills. A qualitative study was conducted in one Australian medical school to investigate the perceptions of students and facilitators involved in teaching and learning clinical communication skills. This study was designed to explore the extent to which communication skills teaching and learning was valued by both students and facilitators; and the extent to which communication approaches taught, aligned with actual clinical practice and students’ perceptions of practice.

## 2. Methods

A qualitative descriptive approach (36) was conducted in a 5-year undergraduate medical program in Australia. This study did not intend to explore the cultural aspect of the phenomenon or explore its lived experience. Instead, the qualitative descriptive approach is suitable for understanding the participant’s perceptions, preferences and feelings about a particular topic (36). Fourth-year medical students and their facilitators were invited to take part in focus group discussions and interviews, respectively. Students’ perceptions and experiences regarding communication skills teaching and learning was explored and compared to the facilitators’ perceptions. The study was approved by the institutional Human Research Ethics Committee.

The communication skills curriculum in this medical program is based on the Calgary-Cambridge Guide to the medical interview (13). This guide is a well-known approach to teaching and training clinical communication skills for health professions education. This model structures medical interview and identifies six essential communication skills tasks, including initiating the session, gathering information, providing structure, building relationships, explanation and planning, closing the session, and overall performance in interpersonal communication (13).

### 2.1. Participant recruitment

The participants were recruited using non-probability sampling, applying a combination of convenience and snowball methods (37). In this sampling method, the participants are selected by criteria defined by the researchers and not calculated using a statistical method. The convenience method allows to include a targeted set of people with similar characteristics and experiences (37, 38). In the context of this study were fourth-year medical students and facilitators who were involved in teaching communication skills. When combining this method with the snowball method, the researcher asks the first few participants to suggest anyone with similar criteria who can participate in the research, and this process results in a wide range of participants who can be approached (37). This recruitment method is commonly used in qualitative studies and is suitable for this study.

All participation was voluntary; the participants were also advised that they could withdraw from the study at any time. Whether or not they decided to participate, this decision would not disadvantage them. The participants were assured that the study would not impact the students’ study progress or the facilitators’ work. The researcher also ensured the participants’ anonymity and confidentiality throughout the conduct of the research project. This information was stated in the participants’ information statements.

In the clinical environment, teaching communication skills is part of clinical supervision, and facilitators do not address communication skills separately (39). Therefore, eligible facilitators were experienced in teaching or supervising pre-registration medical students in the clinical environment (but not necessarily teaching communication skills) and/or teaching or facilitating classroom-based sessions on communication skills teaching with pre-registration medical students for at least 12 months regardless of gender, age, teaching, and clinical experiences. Those who only taught allied health professionals and/or postgraduate students were excluded.

The researchers sent an invitation email to eligible facilitators to participate in a semi-structured interview to share their insight on communication skills teaching and followed up with a reminder to non-responders 2 weeks later. The researcher also contacted suggested facilitators by phone or in person to explain the study and seek expressions of interest to participate. This recruitment process allowed for sampling of facilitators who represented a range of demographic and experience characteristics.

All year-4 medical students were invited to participate in this study during their 12-week Women’s, Adolescent’s, and Children’s Health (WACH) rotation, which involves clinical rotations across five clinical schools in the medical school footprint. All students enrolled in the course during the period of study were invited to participate. The WACH rotation includes learning conducted in the classroom and clinical environments, including clinical communication skills. Students have opportunities to apply skills similar to those covered in classroom modules in the clinical environment. As part of this rotation, students attend four classroom workshops focusing on communication skills required in specific situations. They are also expected to keep a record of experience and achievement toward their core clinical competencies, including history-taking and patient communication tasks. Clinical skills are assessed in a multiple-station OSCE at the end of semester.

All eligible students received an email invitation through the school administration at the beginning of the rotation without the restriction of gender, age, type of enrolment, educational background, or prior work experiences to ensure the credibility of this study. A researcher also attended a compulsory lecture during the introductory common week to elucidate the study and seek expressions of interest to participate. The students were invited to attend a focus group to share their views on communication skills teaching and learning, as well as their experience in practicing clinical communication skills.

### 2.2. Data collection

A researcher interviewed all facilitators to explore their teaching practice and perceptions of students’ communication skills in both the classroom and clinical environment. A semi-structured interview allows researchers to explore interviewees’ responses by asking further questions, enabling in-depth discussion, incorporating new information, and following new ideas as they come up in the interview (38). A semi-structured interview was scheduled for a convenient time and location of the participants’ choosing and could be conducted face-to-face or over the phone.

The interview guide was developed by the research team with expertise in communication skills teaching and learning. Facilitators were invited to briefly talk about their background, experiences, and previous training in medical student teaching, particularly regarding communication skills. They were also asked their views about how different communication skills teaching settings influenced the students, and whether communication skills learnt in the classroom were applied and modeled in the clinical environment, and assessed appropriately. All participants were asked the same questions with slight variations to enable additional exploration of topics as needed, to encourage depth of discussion. All interviews were conducted from August until November 2018 and audio-recorded for transcription purposes.

Students who agreed to participate were invited to take part in focus group discussions. The focus group discussions were designed using guidelines developed by Morgan et al. (40), and a protocol using similar questions as those used for the facilitators was used to guide discussions with students. Focus group discussions collect data from multiple participants simultaneously (40). This method is helpful in exploring participants’ knowledge, experiences, feelings, attitudes and behaviors, as well as the inter-relational dynamic among participants, to enhance the depth and breadth of ideas shared (38, 40). The interactions among participants during discussion could accentuate the similarities and differences and provide richer information about the range of perspective and experiences of the students (38).

A researcher acted as the moderator and asked questions or prompted the group, encouraging participants either to contribute their own input or to comment on each other’s experiences and opinions. Another researcher took field notes and interrupted to ask additional questions if necessary.

The focus group discussion explored experiences of learning and practicing communication skills in different learning environments, facilitation of communication skills learning, and the application and modeling of communication skills learnt in classroom and clinical environments. The discussions were conducted at a tutorial room in each local clinical and scheduled for a maximum of 1 h. Where possible, the discussions were held immediately after timetabled teaching sessions to maximize attendance. A light refreshment was provided during the discussion, and students received remuneration for participation (AUD10). The discussions were audio-recorded for transcription purposes. All discussions were conducted from September until October 2018.

### 2.3. Data analysis

The audio files from interviews and discussions were transcribed by a professional transcribing service, with file security maintained by sharing through OwnCloud. The transcripts were sent to each participant to review for completeness and correctness in capturing what they shared in the discussion. None of the participants objected to the transcripts, and they were then imported to NVivo version 12 for thematic analysis.

Data collected from focus group discussions and interviews were coded thematically to a coding scheme that describe how clinical communication skills were taught, learned, and assessed in different contexts. The scheme was applied to all data with a frequent discussion between the researchers to ensure agreement and consistency in coding. This iterative process was conducted to find patterns and form an understanding of content. Data analysis was synchronous with the data collected, and data were collected to a point where no new codes or categories arose (38). After four focus group discussions and 12 individual interviews, the same information came out repeatedly, and no new information arose. The researchers were satisfied that data saturation had been achieved.

The analysis of interview and focus group data was accompanied by a reflection on how these themes might influence the research questions and findings, as well as the theoretical focus of the study. One researcher performed a preliminary analysis, with the remaining authors acting as second coders for the data. All researchers acted as auditors to cross-check and evaluate the consistency of the code and themes identified. Subsequently, the initial coding was reviewed and compared. It was then contemplated and refined until a consensus was achieved among all researchers, which led to a more representative coding scheme, sub-themes, and themes. The discussion of the identified themes and groupings supported the validity of the coding and developed a thematic framework. A comparison between this thematic framework and the primary raw data was then conducted to ensure that none of the original codes had been excluded mistakenly and that no contradictory data had been dismissed. The analysis process comparing data from student group discussions and facilitator interviews contributed to a triangulation process as a constant comparison to ensure the credibility of the findings and relevance to the research questions. The main themes are summarized with relevant quotes in the results to illustrate findings.

## 3. Results

### 3.1. Descriptive information of the participants

Of the 30 facilitators invited, 13 (43.3%) agreed to be interviewed. One participant decided to withdraw after being interviewed because of feeling they did not have enough experience in medical teaching; therefore, 12 interviews were analyzed. Most interviews were conducted face-to-face, with two conducted over the phone, and each interview lasted between 20 and 30 min (average 28 min and 45 s).

Of the 64 students in one WATCH rotation, 16 agreed to participate in four individual group discussions, each involving between three and six students. Discussions lasted between 45 min and 1 h (average 52 min and 13 s) and were conducted in tutorial rooms in three local clinical schools. Tables 1, 2 provides demographic information of the participants.

**Table 1:** Demographic information of the facilitators.

	Facilitator	
Gender		
Females	6	50.00%
Males	6	50.00%
Age (years)	54.3 ± 8.1 years old (42.0–67.0)	
Years of teaching medical student	19.83 ± 10.50 years (2.4–38.0 years)	
Involvement in teaching		
Course coordination	2	
Curriculum development	2	
Curriculum evaluation	2	
Lecture delivery	5	
Tutors in small group discussion	8	
Facilitation of interactional skills workshops	11	
Clinical supervision	7	
OSCE examination	10	
Moderation of student-lead discussion	4	
Role		
Classroom only facilitators	4	33.30%
Clinical only facilitators	1	8.30%
Both roles	7	58.30%
Educational background		
Pediatricians	4	33.30%
Obstetrics and Gynecology	2	16.70%
ENT surgeon	1	8.33%
Anesthesiologist (ICU specialist)	1	8.33%
Social worker	1	8.33%
Health behavioral scientist	3	25.00%

**Table 2:** Demographic information of the students.

	Student	
Gender		
Females	10	62.50%
Males	6	36.50%
Age (years)	25.0 ± 5.8 y.o (21.0–46.0)	
Educational background		
Secondary high school	15	93.75%
Undergraduate	1	6.25%
Student previous work in the health field		
None	13	81.25%
Aged care	1	6.25%
Ambulance crew	1	6.25%
Others (not specified)	1	6.25%

### 3.2. Perception of students and facilitators

Primary themes included the value of communication skills teaching and learning, alignment between approaches to teaching and actual clinical practices and students’ perceptions of practice, the importance of feedback, and the challenges in different environments. Themes and sub-themes are described below, with illustrative quotes from facilitators [F] and students [S].

#### 3.2.1. The value of teaching and learning communication skills in the classroom

Both students and facilitators valued classroom teaching and learning (known as interactional skills sessions) as a safe space to practice and learn the framework of patient consultations. This interactional skills session was a small group session of 10–12 students, facilitated by a classroom facilitator and a simulated patient. Students appreciated the structure, which gave them the confidence to engage with real patients and adapt appropriately.

*I think interactional skills definitely make me more comfortable with patients and kind of having that framework beforehand to sort of go back on.* [S8]

*I feel like having a checklist and having a goal in mind before you actually interact with the patient*. [S2]

Students felt that classroom learning helped them become critical observers of what they saw in clinical practice.

*Some of these sessions have given [me] a lens through which to observe and pick up on the skills*. [S12]

*Sometimes you see bad examples being applied to the real world, and I am glad we have these interactional skills sessions because that tells you what good and bad examples are* [S3].

While students viewed the classroom positively, several classroom facilitators felt that students did not value the sessions. Students’ preparation and willingness to volunteer for role-playing were two main concerns.

*They have not read the pre-reading package, so they quite often struggle with being able to participate*. [F13, classroom-only facilitator]

*I know that some students really hate the role play, so they do not want to do it*. [F5, classroom-only facilitator]

The small number of students in an interactional skills session led to more interactive session. However, some students reported that being the center of attention during a role-play was daunting or challenging, and it was this which prevented them from volunteering. However, the safe practice opportunities were valued, as one student noted:

*For some reason, I get really anxious about participating (in role-play) and so I never volunteer. But I find the sessions so much more useful if I participate*. [S11]

Students said that by observing other students doing role-play, they also benefited by learning different approaches to communication.

*Just observing your peers communicating with the same patient helps you in a way it allows you to see the variety of approaches that each individual student takes to the patient*. [S2]

*Observing your peers helps to modify your technique of communicating with the patient*. [S3]

### 3.3. Alignment of approaches to teaching with actual clinical practice and students’ perceptions of practice

In clinical environments, students learned communication mainly through observing clinicians. Students had experience talking with real patients; however, in the absence of being observed by clinicians, the learning value was potentially limited.

*It would be helpful if there were doctors who actually observed us while we spoke to the patient*. [S2]

*The staff, like myself actually, are taking the medical history ... So, we do not ask the student to take the history… I do not often see them interacting with client or patient...* [F12, classroom & clinical facilitator]

Students felt the role of facilitators in teaching communication skills, both in the classroom and clinical environments, was crucial. The value of each session depended on the facilitators’ approach. The clinical facilitators provided examples of how to apply the skills during a patient encounter, and students saw this as validating classroom learning. For that reason, students preferred having facilitators that taught medical content related to clinical practice and tips on passing the assessment.

*Obviously, you want a facilitator that knows their stuff*. [S2]

*I think the sessions have been heavily dependent on how the tutor runs the session*. [S9]

All facilitators had done some faculty development programs for general teaching. In addition, the clinical facilitators had completed communication skills training as part of their own medical and residential training. While the classroom-only facilitators had participated in training specifically for teaching communication skills, none of the clinical facilitators (most of whom also taught in the classroom) had done so. Few had knowledge of the medical curriculum beyond the component they taught.

*No formal teaching by the university, but mostly informal training*. [F5, classroom and clinical facilitator]

*I do not know what they get in the classroom, and I only know the content that I teach*. [F10, clinical-only facilitator]

Those teaching in the clinical environment were aware that their interaction with patients influenced students’ communication skills. Despite this, none of the clinical facilitators related their communication skills to a particular model of consultation. Rather, they based their communication with patients, as well as their teaching on their experiences in clinical practice.

*It (the consultation model) is based on two decades of personal experience … I am not familiar with the Calgary communication model*. [F10, clinical-only facilitator]

*I think most of us (clinicians) do not model the ones that we teach the students and want them to do*. [F11, classroom and clinical facilitator]

Students also reported that different clinical disciplines focused on specific aspects, which was sometimes contrasted with what they had learnt about a holistic approach and comprehensive history-taking. Some clinical facilitators confirmed this finding, emphasizing the varied value of communication skills in different disciplines.

*Depends on the specialty … in some medical skills, we are taught to have a holistic approach …, but in the surgical ward, you need only to care about the surgical issues*. [S1]

*You go to your surgeon, and he spends half an hour explaining that he is going to put a plate in your broken leg, and he does a lousy job. Would you prefer him or a surgeon who just puts the plate in, and you know it is going to be good?* [F8, classroom and clinical facilitator]

*There is only a handful of us in this course are involved in the interactional skills session. So, I am sure the vast majority of people who are supervising the students, probably do not have much contact*. [F11, classroom and clinical facilitator]

### 3.4. The importance of feedback on communication skills learning

Both facilitators and students agreed that feedback helped develop student’s communication skills and increased their confidence to talk with real patients in clinical environments. The classroom facilitators provided feedback on the structure and process skills of communication, including the use of open-ended questions, medical jargon, and non-verbal behavior when students role-played in an interactional skills session. In the classroom, the feedback was provided by inviting the students to self-evaluate and inviting peers and simulated patients to give feedback as well.

*I focus on the kind of language they used, the pace at which they speak, the body language, all the different kinds of modalities of communication*. [F7, classroom and clinical facilitator]

*I tend to use the ALOBA model, when before beginning, a role-play with a simulated patient, I will ask the students what they would like the feedback on from myself and their peers*. [F6, classroom-only facilitator]

The clinical facilitators described receiving reports of patient medical histories from students rather than directly observing students conducting consultations. Therefore, feedback primarily focused on the medical history information obtained and clinical reasoning instead of how to interact with patients. Students confirmed that they were rarely observed during patient encounters but said they would value feedback on how they communicated with patients.

*We rarely provide feedback in the outpatient setting …But in the interactional skills session, the feedback might be about their communication skills*. [F11, classroom and clinical facilitator]

*If they take a history with a patient and they come and present to me, then I can correct, “You did not do this. You did not ask this.”* [F9, classroom and clinical facilitator]

*The feedback that you get from the sim patients and from the clinician who is observing you, I think that is really useful because you do not get that in the real-world setting*. [S15]

### 3.5. Challenges of communication skills learning in different learning environments

While both students and facilitators appreciated the classroom and clinical environment, there were some challenges to applying their skills in clinical practice.

#### 3.5.1. Realism in the classroom

Both facilitators and students felt that role-plays used in the classroom and assessment setting were artificial and “far from realism” [S1]. For example, students reported that while the actors could portray patients well, as they came to know the regular actors, realism was compromised.

*Because the simulated patients do not behave and react like a real patient. ... Whilst you can pretend you have lost a child or you can pretend you presented with bleeding in pregnancy, it is not the same as when it happens to you*. [F10, clinical-only facilitator]

*A few weeks ago, we had the ‘talking to adolescents’ session. We found it was difficult for an adult (simulated patient) to relate to teenagers when we were close to that*. [S10]

#### 3.5.2. Participation in the clinical environment

The students felt that the clinical environments provided opportunities to apply their skills. However, busy clinical environments and time pressures in the hospital limited the opportunities to talk with the patients and be observed by and receive feedback from the facilitators. The facilitators echoed these sentiments.

*I knew I needed a whole bunch of time to speak to a patient about her son's condition, but in the real world, what if there were five other patients that had really urgent (issues)?* [S1]

*When they (students) come to our standard clinic, there is not usually available time to watch or discuss with the students*. [F11, classroom & clinical facilitator]

During clinical rotation, students were part of a clinical team, including a range of health professionals. The facilitators mentioned that the task hierarchy in this system might limit opportunities for the students to talk with patients.

*They are often intimidated by the fact that they are part of a group that includes junior doctors, middle-ranked doctors, senior doctors, consultants, and senior nurses*. [F8, classroom and clinical facilitator]

#### 3.5.3. Learning for assessment

Facilitators reflected that some students seemed to only focus on completing requirements to pass the assessment. They were concerned that students were missing opportunities to develop rapport and understand patients’ psychosocial situations during assessments.

*At the moment, I think a lot of their direct one-on-one patient interactions relate to their specific testing and their other forms that they need to get done*. [F10, clinical only facilitator]

*I think that (assessment) can sometimes be a bit narrow in terms of the students can be very ‘point-wise,’ just trying to tick the boxes and getting all the information, but not really going into details*. [F12, classroom and clinical facilitator]

Some students also said they were more motivated to pass their examination than developing their counseling skills.

*Because our main concern is just passing the OSCE and to just tick whatever boxes I need to tick off*. [S3]

*You become so – what is the word – ‘entangled’ in dealing with just fulfilling the checklist*. [S2]

Facilitators mentioned limitations of OSCE assessments which could perpetuate this challenge as well the realism of these assessments.

*We have an OSCE of giving somebody news like telling them a fetus is dead. But they only give 8 min to explain it. This sends a bad message to the students that they can do this*. [F5, classroom-only facilitator]

*We are not currently assessing this particular topic – I do not think we have an OSCE scenario, for example, around child protection*. [F2, classroom & clinical facilitator]

## 4. Discussion

### 4.1. Main findings

This study reinforces the value placed on teaching and learning clinical communication skills by facilitators and students. Both groups agree that classroom learning provides students with a structure to use in communicating with real patients, which can be modified to suit various situations and patient needs. Though, the lack of realism and the discomfort of being the centre of attention during role-play was seen by students as disadvantages of this type of learning. Despite these challenges and facilitators’ perception of students’ lack of engagement, these learning opportunities were valued by students, particularly in providing a structure to apply in interactions with real patients and in observing clinicians. Students have limited opportunities, however, to be observed and receive feedback on their real-patient encounters.

The facilitators and clinical environment are the main factors contributing to the effectiveness of clinical communication skills teaching. However, discrepancies exist in the educator training and approaches used between the classroom and clinical facilitators. Discrepancies of communication skills teaching between classroom and clinical practice found in this study are also reported in previous research (41, 42). While in the classroom, students are observed and receive feedback on the process of their communication skills, in the clinical environmental unstructured teaching, less observed and feedback focuses on clinical knowledge is reported.

Students agreed that they also learned clinical communication skills by observing doctors in their own practice. During teaching in the clinical environment, communication issues are rarely specifically highlighted. They are usually only addressed if students have ethical issues or specific difficulties, such as talking with children or breaking bad news (18). History taking and physical examination are commonly taught by specialist, while communication issues are more often discussed by general practitioners, psychiatrists, and health behavior scientists (43). This partitioning of skills sends a ‘hidden’ message to students that many clinicians do not value communication skills, and their application is not needed for quality healthcare. This hidden curriculum can play a significant role in shaping students’ own behaviors and attitudes (44, 45). Findings in this study support previous evidence that communication skills modeled by clinical facilitators differ from what students learn in the classroom (29). The recognition of learning through observation made by the students in this study is also in keeping with previous research (46) and is problematic given the discrepancy between best practice and that which is modeled.

The educational conditions in clinical environments are often considered not ideal for learning, particularly the lack of supervision and chaotic environments (47). The value of patient encounters in the clinical environment is likely to be limited in the absence of direct observation and feedback, as reported in this study. These findings are in keeping with previous literature demonstrating that encounters with real patients facilitate the transfer of learning into the workplace (20, 48). However, without observation and feedback during clinical encounters, communication skills may deteriorate during clinical rotations (49).

Both participant groups believed that facilitators played a crucial role in clinical communication skills teaching, as supported by previous studies (11, 20, 39). On the other hand, studies also find that clinicians are juggling between their teaching and other professional roles (50, 51). Thus, time constraints and poor modeling in clinical environments can cause students to focus on clinical content elements rather than the process skills inherent in effectively gathering a patient history (52). Still, clinicians are often preferred as facilitators for clinical communication skills teaching, as reported by students in our study, due to their ability to link theory to clinical practice.

While students in this study were educated and assessed based on the Calgary Cambridge model of the medical consultation (13), clinical facilitators could not identify a particular model upon which their consultation practice or teaching was based. Instead, they based their teaching on their years of clinical experience. Briefings to prepare clinical facilitators for clinical communication skills teaching are offered by the medical faculty in which this program is situated. However, the clinical facilitators rarely take up these opportunities or engage with the curriculum beyond their own area. Difficulties engaging clinicians in faculty development, mainly related to their roles as educators, have been previously documented (53), and the availability of training specifically for communication skills teaching is scarce (54). This situation reflects the difficulty in actively engaging clinicians in teaching-related activities in behavioral sciences and communication skills.

A review of interventions to improve medical students’ communication skills reports that tailored, specific feedback shows the most significant promise in terms of influencing students’ communication skills (16). The clinical facilitators emphasized the importance of rapport-building during patient consultations but only provided feedback on students’ rapport building in the classroom setting where students are observed in their interactions with simulated patients. In clinical environments, feedback is primarily focused on content and clinical reasoning for patient management (17). This situation perpetuates the dichotomy between process and content that causes students to confuse which to focus on real patient interaction, whether the medical content or patient-centeredness (49). This dichotomy is reported as a primary challenge for clinical communication skills teaching (15, 18), as also reported by the students in this study.

Our study focuses on undergraduate medical students, while teaching clinical communication skills for health professions also becomes an issue. The principle of experiential learning and feedback is similar; however, flexible training to adapt to clinical workloads, local needs, and circumstances is the main concern in communication skills training for health professions (55). In our study, clinical facilitators involved in teaching medical students are untrained to communication skills. Instead, in health professions, trained clinical facilitators with an understanding of communication skills curriculum, the skills, able to observe interaction and provide feedback and reflection are crucial (55). This again shows the complexity of communication skills which entails the development of clinical and human skills and should be conducted as part of lifelong learning.

### 4.2. Implication for practice

A combination of classroom simulation and real-patient encounters is the preferred approach to learn clinical communication skills (15). However, finding the ideal balance between the two learning environments and the volume of practice is an ongoing challenge. By combining formal communication skills in classroom sessions with the learning occurring during clinical rotations, as well as being observed and receiving feedback, students can balance both content and process of communication skills. Furthermore, the classroom sessions during clinical rotations offer the opportunity for students to discuss and reflect on their experiences (18). However, the success of this relies on facilitators with sufficient skills to facilitate communication skills learning and providing feedback. Therefore, training for clinical facilitators involved in teaching medical students should become a comprehensive agenda to ensure continuous clinical communication skills training in every step of medical education.

### 4.3. Limitation of study

This study has several limitations. First, this study was conducted in a single institution, inviting all facilitators of relevant interactional skills classroom sessions and clinical rotations to participate. Almost all clinical facilitators interviewed also taught in the classroom. These participants are likely to have more awareness of the communication skills issues discussed, models of the medical consultation, and approaches to teaching, than those who do not engage in classroom teaching. The finding that even among this group, awareness of the curriculum and consultation models was low, and observation of students’ communication with patients was infrequent, suggests these practices are even less common in the clinical teaching population as a whole.

Secondly, this study employed convenient and snowball methods to recruit participants. This study was also voluntary, and students who participated might have stronger motivation to learn. Medical students from other programs or different years of study might have had different experiences. Therefore, the generalization and transferability of findings might be restricted. However, the random nature of the clinical schools and rotation allocations, as well as the demographic spread of participants suggest that a relatively representative sample was obtained.

Furthermore, this study combined data from two data collection methods, interviews and focus groups. Integration of data from different data collection methods may threaten the trustworthiness of the findings (56). Though combining different methods in this study offered different perceptions and experiences of students and facilitators and enriched the findings of this study.

## 5. Conclusion

This study improves the understanding of the value of communication skills teaching and learning in the classroom as preparation for clinical environments. The two learning environments complement each other with their strengths and weakness. While the classroom offers a safe place for learning and the availability of feedback, the clinical environment offers opportunities to experience clinical communication skills in real practice. The critical components of role-play practice, feedback, observation, and supervision are well-acknowledged, but the quality of application of each of these components differs across learning environments. Teaching and learning communication skills using a combination of classroom and clinical environments during clinical rotation is recommended to strengthen the learning of both the content and process of communication skills. The role of clinical facilitators in communication skills teaching requires more engagement and faculty development programs, as well as organizational support to enable facilitators to observe and provide feedback on real-patient encounters and model skills consistent with those being taught in the classroom.

## Data availability statement

The raw data supporting the conclusions of this article will be made available by the authors, without undue reservation.

## Ethics statement

This study involving human participants was reviewed and approved by the University of Newcastle Human Research Ethics Committee (HREC #H-2018-0152). The participants provided their written informed consent to participate in this study.

## Author contributions

SD, CG, and AW contributed to the conception and design of the study and analyzed the data. SD organized and did data collection and pre-analysis and wrote the first draft of the manuscript. All authors contributed to manuscript revision, read, and approved the submitted version.

## Funding

This study was funded by the Higher Degree Research Fund from the University of Newcastle, Australia. The authors gratefully acknowledge the Universitas Padjadjaran for the financing for the publication in Open Access.

## Conflict of interest

The authors declare that the research was conducted in the absence of any commercial or financial relationships that could be construed as a potential conflict of interest.

## Publisher’s note

All claims expressed in this article are solely those of the authors and do not necessarily represent those of their affiliated organizations, or those of the publisher, the editors and the reviewers. Any product that may be evaluated in this article, or claim that may be made by its manufacturer, is not guaranteed or endorsed by the publisher.
